# Molecular cytogenetics of valuable Arctic and sub-Arctic pasture grass species from the *Aveneae*/*Poeae* tribe complex (*Poaceae*)

**DOI:** 10.1186/s12863-019-0792-2

**Published:** 2019-12-04

**Authors:** Alexandra V. Amosova, Svyatoslav A. Zoshchuk, Alexander V. Rodionov, Lilit Ghukasyan, Tatiana E. Samatadze, Elizaveta O. Punina, Igor G. Loskutov, Olga Yu. Yurkevich, Olga V. Muravenko

**Affiliations:** 1 Engelhardt Institute of Molecular Biology, Russian Academy of Sciences, Moscow, Russian Federation; 2 Komarov Botanical Institute, Russian Academy of Sciences, St. Petersburg, Russian Federation; 3 Federal Research Center N.I. Vavilov All-Russian Institute of Plant Genetic Resources (VIR), Russian Academy of Sciences, St. Petersburg, Russian Federation

## Abstract

**Abstract:**

**Background:**

Grasslands in the Arctic tundra undergo irreversible degradation due to climatic changes and also over-exploitation and depletion of scarce resources. Comprehensive investigations of cytogenomic structures of valuable Arctic and sub-Arctic grassland species is essential for clarifying their genetic peculiarities and phylogenetic relationships, and also successful developing new forage grass cultivars with high levels of adaptation, stable productivity and longevity. We performed molecular cytogenetic characterization of insufficiently studied pasture grass species (*Poaceae*) from related genera representing two neighboring clades: 1) *Deschampsia* and *Holcus*; 2) *Alopecurus*, *Arctagrostis* and *Beckmannia*, which are the primary fodder resources in the Arctic tundra.

**Results:**

We constructed the integrated schematic maps of distribution of these species in the northern, central and eastern parts of Eurasia based on the currently available data as only scattered data on their occurrence is currently available. The species karyotypes were examined with the use of DAPI-banding, multicolour FISH with 35S rDNA, 5S rDNA and the (GTT)_9_ microsatellite motif and also sequential rapid multocolour GISH with genomic DNAs of *Deschampsia sukatschewii*, *Deschampsia flexuosa* and *Holcus lanatus* belonging to one of the studied clades*.* Cytogenomic structures of the species were specified; peculiarities and common features of their genomes were revealed. Different chromosomal rearrangements were detected in *Beckmannia syzigachne*, *Deschampsia cespitosa* and *D. flexuosa*; B chromosomes with distinct DAPI-bands were observed in karyotypes of *D. cespitosa* and *H. lanatus*.

**Conclusions:**

The peculiarities of distribution patterns of the examined chromosomal markers and also presence of common homologous DNA repeats in karyotypes of the studies species allowed us to verify their relationships. The obtained unique data on distribution areas and cytogenomic structures of the valuable Arctic and sub-Arctic pasture species are important for further genetic and biotechnological studies and also plant breeding progress.

## Background

According to current molecular phylogenetic studies, the tribe *Poeae* R. Br. (*Pooideae, Poaceae*) now includes the formerly separate tribe *Aveneae* Dum [1, 2]. The *Aveneae*/*Poeae* tribe complex comprises a large number of crops and forage plants which play an important role for humans and animals. As an example, economically important oat is the most ancient food supplies for humankind [3]. Lots of members of the *Aveneae*/*Poeae* tribe are polymorphic species with a wide geographical distribution, high morphological diversity and complicated taxonomy [1, 2, 4, 5]. Species of this tribe are highly tolerant to stressful and variable environmental conditions including extreme Arctic and Antarctic habitats [6–8]. In particular, *Deschampsia antarctica* E. Desv. is the only one species within the *Poaceae* family adapted to the harshest Antarctic environment [7]. Polar and subpolar ecotypes of perennial cereals from the related genera *Alopecurus*, *Arctagrostis*, *Beckmannia*, *Deschampsia* and *Holcus* are characterized by high biological value, stable productivity and longevity and used as native and/or introduced forage resources in the Arctic tundra [6, 7, 9–12]. Animal husbandry has always been the principal economic activities of the northern indigenous peoples (the Yakuts, Nenets, Evenki, etc.), and pasture grasses are the primary fodder resources in the Arctic tundra [10]. Currently, the unique Arctic ecosystem requires special attention as grasslands in the Arctic regions undergo irreversible degradation due to climatic changes and also over-exploitation and depletion of scarce resources [10]. Sustainable grazing strategies can have a significant impact on rehabilitating the degraded grasslands. Also, introduction of native Arctic grassland species and also non-native cold-hardy ecotypes of perennial grasses with high levels of adaptation and seed productivity as well as development of new valuable cultivars from promising wild morphotypes could be the strategies for reducing feed costs and increasing feed efficiency in the Arctic and sub-Arctic regions [11, 12]. Moreover, the investigation of the evolutionary changes occurred in their genomes (especially, under environmental stress factors) is an important aspect for clarifying the mechanisms related to abiotic stress tolerance and for further crop breeding strategies [13, 14].

Despite widespread occurrence and economic value of most cereals from the *Aveneae*/*Poeae* tribe, their genomes still remain insufficiently studied [14, 15]. Due to the agricultural significance, oat species have become the subject of complex investigation, and extensive molecular studies have largely clarified diversity and evolution of the genus *Avena* [16–20]. Phylogenetic studies in recent decades, mostly based on analyses of different DNA sequences from both plastid regions and nuclear ribosomal regions, have helped to clarify the evolutionary relationships within the *Aveneae*/*Poeae* complex [4, 5, 15, 21–23]. However, the details of the phylogeny among many genera and suprageneric taxa of this diverse grass tribe still remain controversial [23]. Furthermore, the complicated breeding system, that is peculiar to evolution of major cereals from this tribe, included interspecific and intergeneric hybridization which was accompanied by the allopolyploid formation [24]. Moreover, environmental stress factors can lead to physiological stress followed by structural variations (chromosome rearrangements, mixoploidy and aneuploidy) in plant genomes [25–27]. Therefore, the investigation of genomes of such species should comprise their molecular cytogenetic characterization [15, 23]. Particularly, physical mapping of specific DNA probes to individual chromosomes is a promising tool for analysis of chromosome organization and evolutionary processes in plant genomes [15]. Knowledge of the chromosome localization of repetitive DNA families is very useful in the assembly of genome sequences and their assignment to physical chromosomes [28].

Currently, detailed cytogenetic information is available for less than half of the altogether 60–70 genera of the *Aveneae*/*Poeae* [15, 29], and among them, the genus *Avena* is the most extensively studied with the use of FISH with different sat DNAs and staining of constitutive heterochromatin regions on chromosomes [30–34]. Molecular phylogenetic and cytogenetic studies indicated that diploid *Avena* species carried different types of the A genome (As; Al; Ac; Ad and Ap) or C genome (Cv and Cp); tetraploid *Avena* species (2n = 4x = 28) comprised AB or AC genomes; and hexaploid species (2n = 6x = 42) had ACD genomes [16–20, 30–32]. Also, it was assumed that Al genome was the most ancient oat genome which could be a possible progenitor of B and D genomes of the genus *Avena* [16–20, 30–32, 35]. Besides, molecular cytogenetic data were reported for several taxa of the *Aveneae*/*Poeae* genera including genera *Arrhenatherum*, *Deschampsia*, *Helictotrichon*, *Pseudarrhenatherum*, *Trisetum* and some others. Particularly, for *D. cespitosa* (L.) P. Beauv., a chromosome number 2n = 26 = 2(6 m + 4sm + 3st) with three pairs of satellite chromosomes was determined and also chromosomal distribution of rDNA sites and CON/COM DNA repeats were described [15, 36]. Recently, we have performed a comparative molecular cytogenetic analysis of several *Deschampsia* species sampled in different regions and environments including sub-Arctic mountain tundra (*D. sukatschewii* (Popl.) Roshev) [27, 37]. FISH with rDNA and rapid GISH with genomic DNA of closely related (*D. antarctica* E. Desv. and *D. cespitosa*) and also the most distant (*D. flexuosa* (L.) Trin. (=*Avenella flexuosa* (L.) Drejer) species of the genus have indicated variations in the karyotype structure and ploidy status as well as in distribution of the studied chromosomal markers, which could be related to plasticity of their genomes [27, 37].

Nevertheless, cytogenetic data on most members of the *Aveneae*/*Poeae* tribe still remain limited; in particular, karyotypes of many valuable pasture grass species important for Arctic and sub-Arctic regions (including *Alopecurus aequalis* Sobol. (2n = 14), *Alopecurus arundinaceus* Poir. (2n = 4x = 28), *Arctagrostis latifolia* (R. Br.) Griseb. (2n = 4x = 28), *Beckmannia syzigachne* (Steud.) Fernald*.* (2n = 14) and *Holcus lanatus* L. (2n = 14)) have been analysed by simple monochrome staining which resulted in the determination of chromosome number and ploidy level in these species [35, 38].

### Objectives

In this study, we performed a molecular cytogenetic characterization of sub-Arctic accessions of *A. aequalis*, *A. arundinaceus*, *A. latifolia*, *B. syzigachne*, *D. cespitosa*, *D. flexuosa* and *H. lanatus* in order to specify their ploidy status and karyotypic features and also reveal possible structural chromosome rearrangements important for further crop breeding strategies. For a comparative assessment of cytogenetic peculiarities and karyogenomic similarities within the studied species, we also characterized an accession of *Avena longiglumis* Dur. containing the most ancient oat Al genome which also could be useful for development of new forage grass cultivars [12, 35, 40].

## Methods

### Plant material

Seeds of sub-Arctic tundra accessions of *A. arundinaceus* (144, Magadan region, RF: 59°34′ N; 150°48′ E), *A. latifolia* (85, Magadan region, RF: 59°34′ N; 150°48′ E), *B. syzigachne* (93, Magadan region, RF: 59°34′ N; 150°48′ E), *D. cespitosa* (688, Vologda region, RF: 59°13′ N; 39°54′ E), *D. flexuosa* (598, Republic of Karelia, RF, 63°49′ N; 33°00′ E) and a Sub-Arctic mountain tundra accession of *D. sukatschewii* (78, Altai Mountains, 1400 m above mean sea level (mamsl), RF: 48°45′ N; 89°36′ E) were received from the Laboratory of genetic resources of fodder plants, Federal Williams Research Center of Forage Production and Agroecology, Lobnya, Moscow region, RF. Seeds of *A. longiglumis* (k-1811, Morocco) were received from the Department of genetic resources of oat, rye and barley, FRC N.I. Vavilov All-Russian Institute of Plant Genetic Resources (VIR), St. Petersburg, RF. Sub-Arctic mountain tundra accessions of *A. aequalis* (Alt11–309, natural populations, Altai Mountains, 1200 mamsl, RF: 48°45′ N; 89°36′ E) and *H. lanatus* (T-115, natural populations, Caucasus Mountains, 1400 mamsl, RF: 43°46′38″ N; 41°55′00″ E) were sampled and identified by Dr. E.O. Punina, Laboratory of Biosystematics and Cytology, Komarov Botanical Institute, RAS, St. Petersburg, RF. Collection of these wild plant samples and experimental research conducted on these materials were performed in accordance with the legal regulations of Komarov Botanical Institute, RAS, St. Petersburg, RF approved by the Ministry of Science and Higher Education of the Russian Federation.

### Chromosome spread preparation

Mitotic chromosome spreads were prepared from plant root meristem according to the previously described technique [37].

.

### MC-FISH procedure

Following probes were used for MC-FISH:

1. pTa71 containing a 9 kb long DNA sequence of common wheat including 18S-5.8S-26S (35S) rDNA [41]
2. pTa794 containing a 420 bp long DNA sequence of wheat including 5S rDNA [42]

These DNA probes were labelled directly with SpectrumAqua and SpectrumRed fluorochromes (Abbott Molecular, Wiesbaden, Germany) by nick translation according to manufacturers’ protocols.

3. The oligo-(GTT)_9_ probe, labelled at the 3′-end with fluorescein-12-dUTP (Roche diagnostics, Mannheim, Germany), was synthesized using a synthesizer ABI 394 (applied BioSystems, Redwood City, USA)

MC-FISH assays were performed with the use of a combination of these DNA probes as described previously [43]. After overnight hybridization, the slides were washed twice with 0.1xSSC at 44 °C for 10 min, twice with 2xSSC at 44 °C for 5 min followed by a 5-min wash in 2xSSC and three washes in PBS for 3 min each at room temperature. Then the slides were dehydrated through a graded ethanol series and air dried. The oligo-(GTT)_9_ probe labelled with fluorescein was detected using anti-fluorescein/Oregon green, rabbit IgG fraction, Alexa Fluor 488 conjugate (Molecular Probes, Oregon, USA).

### DAPI staining

After MC-FISH procedures, chromosome slides were stained with 0.1 μg/ml DAPI (4′,6-diamidino-2-phenylindole) (Serva, Heidelberg, Germany) dissolved in Vectashield antifade mounting medium (Vector laboratories, Peterborough, UK).

### Rapid MC-GISH procedure

Genomic DNAs of *D. sukatschewii*, *H. lanatus* and *D. flexuosa* were isolated from green leaves using CTAB (cetyltrimethylammonium bromide) standard protocol with minor modifications [44]. Then these genomic DNAs were labelled directly with SpectrumAqua or SpectrumRed (Abbott Molecular, Wiesbaden, Germany) by nick translation according to the manufacturer’s instructions.

After the MC-FISH procedure and documentation of the hybridization patterns, a sequential rapid MC-GISH procedure was conducted on the same slide as described previously [27]. In rapid MC-GISH assays, labelled genomic DNAs of *D. sukatschewii* and *H. lanatus* (for *A. aequalis*, *A. arundinaceus*, *A. latifolia*, *A. longiglumis*, *B. syzigachne*, *D. cespitosa* and *D. flexuosa*) or *D. flexuosa* and *D. sukatschewii* (for *H. lanatus*) were used as DNA probes.

### Chromosome analysis

The chromosome slides were examined with the use of an Olympus BX61 epifluorescence microscope (Olympus, Tokyo, Japan) equipped with a cooled high-resolution black and white CCD camera (Cool Snap, Roper Scientific Inc., Tucson, USA). Chromosome images were collected in grayscale channels and pseudocoloured. Then they were processed with Adobe Photoshop 10.0 (Adobe Systems Inc., Birmingham, USA) and VideoTesT-FISH 2.1 (IstaVideotest, St. Petersburg, Russia) software programs. In each species accession, five plants with fifteen metaphase plates were examined. In the karyotypes, the identification of chromosomes was performed based on their morphological similarities as well as revealed molecular cytogenetic markers. In the species karyograms of *A. aequalis, A. arundinaceus*, *A. latifolia*, *A. longiglumis*, *B. syzigachne* and *H. lanatus*, the chromosome pairs were set in the decreasing order of size. The cytological numerical designation of *D. cespitosa* and *D. flexuosa* was according to Amosova et al. [37].

## Results

### Species distribution areas

**Fig. 1:**
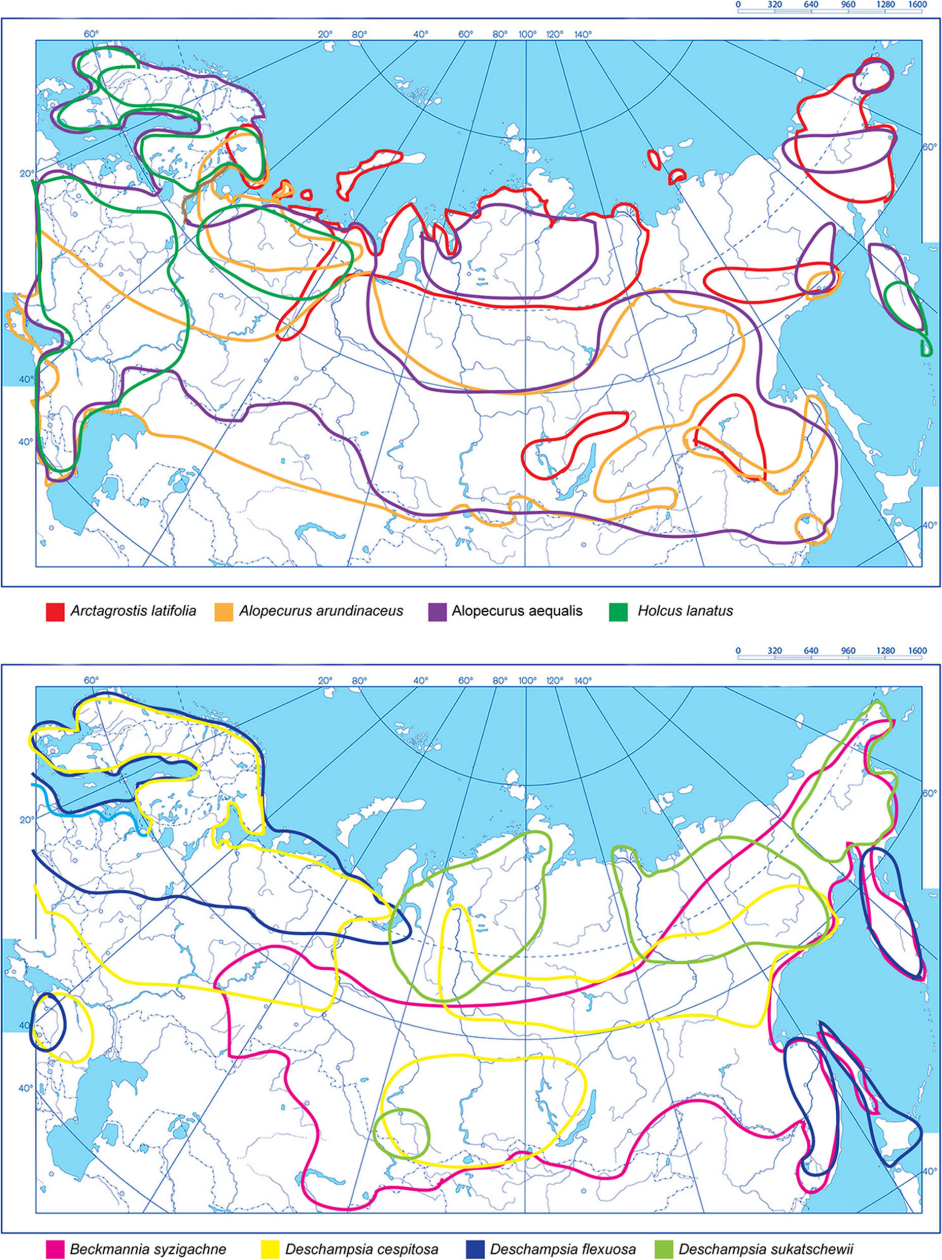
Integrated schematic habitat maps showing distribution of *A. aequalis*, *A. arundinaceus*, *A. latifolia*, *B. syzigachne*, *D. cespitosa*, *D. flexuosa*, *D. sukatschewii* and *H. lanatus* within the northern, central and eastern parts of Eurasia. The species names and correspondent colours of the lines indicating the boundaries of the species occurrence are specified under the maps

For *A. aequalis, A. arundinaceus*, *A. latifolia*, *B. syzigachne*, *D. cespitosa*, *D. flexuosa*, *D. sukatschewii* and *H. lanatus*, we constructed integrated schematic maps of their distribution in the northern, central and eastern parts of Eurasia based on the analysis of currently available data [2, 6, 45–52] (Fig. 1). In these regions, *A. latifolia* is distributed from the Arctic Archipelago of Svalbard to Chukotka Peninsula including the Archipelago of Novaya Zemlya (75° N). Circumboreal-polar species, *A. aequalis*, *A. arundinaceus* and *D. cespitosa* are widespread in the Eurasian continent from the Arctic Scandinavia to the Far East regions and Chukotka Peninsula. Northern populations of *H. lanatus* and *D. flexuosa* occur in the Arctic Scandinavia and Kola Peninsula and also in Kamchatka Peninsula and Far East regions. *B. syzigachne* is widespread in the Eurasian continent from the Volga river basin to Chukotka Peninsula. *D. sukatschewii* is distributed in all northern regions of Siberia and Chukotka Peninsula (Fig. 1).

### Karyotype structure

**Fig. 2:**
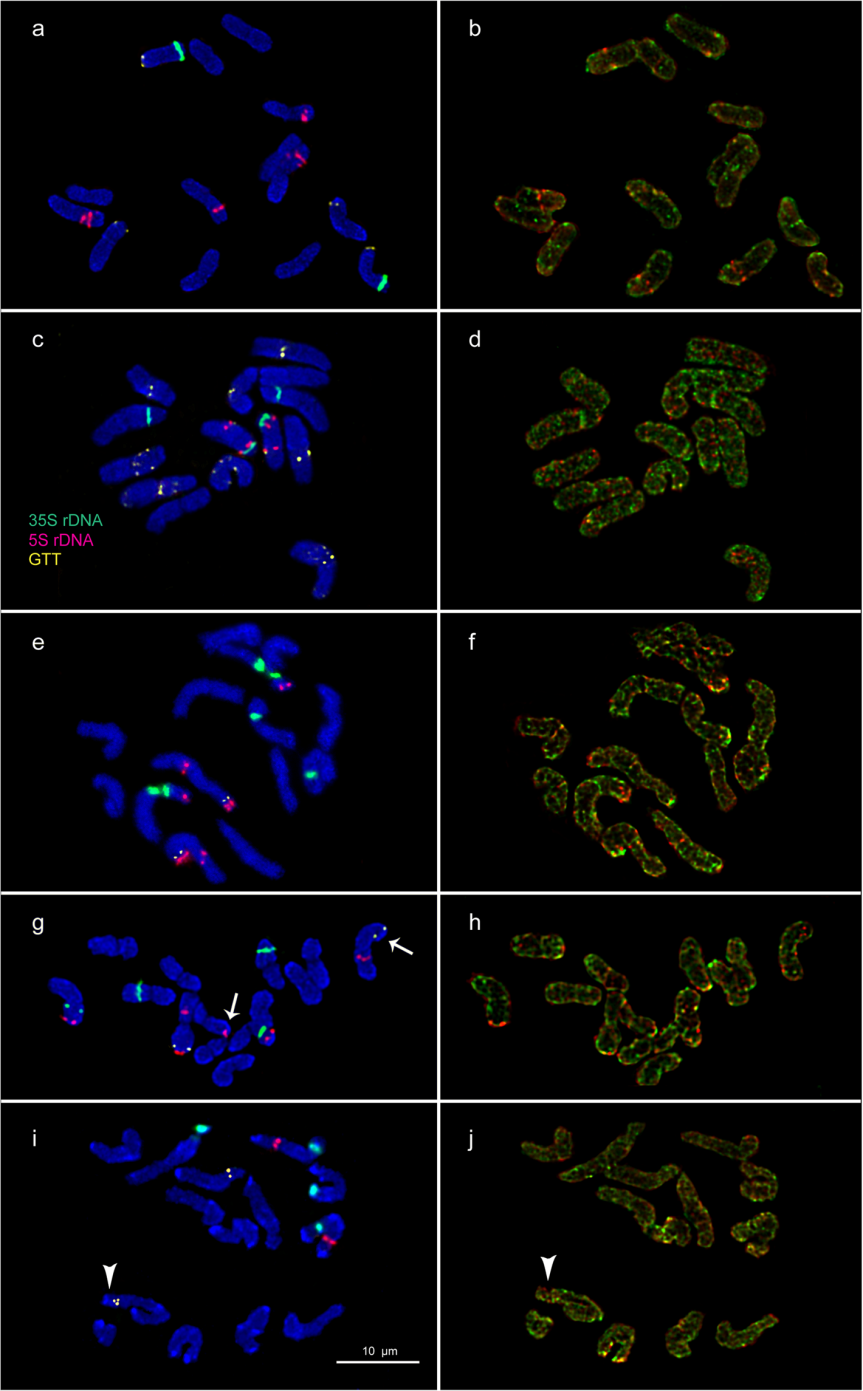
Chromosome spreads of the studied diploid species. **a**, **b** *A. aequalis*, **c**, **d** *A. longiglumis*, **e**, **f** *B. syzigachne* (normal karyotype), **g**, **h** *B. syzigachne* (karyotype with a translocation t(1;6)) and **i**, **j** *H. lanatus*. Merged fluorescent images after MC-FISH with 35S rDNA (green), 5S rDNA (red), oligo-(GTT)_9_ (yellow) **a**, **c**, **e**, **g** and sequential rapid MC-GISH with genomic DNAs of **b**, **d**, **f** *D. sukatschewii* (red) and *H. lanatus* (green) and also **h** *D. sukatschewii* (red) and *D. flexuosa* (green). DAPI chromosome staining - blue. Arrows point to chromosome rearrangements. Head of arrows point to a B chromosome. Scale bar - 10 μm

**Fig. 3:**
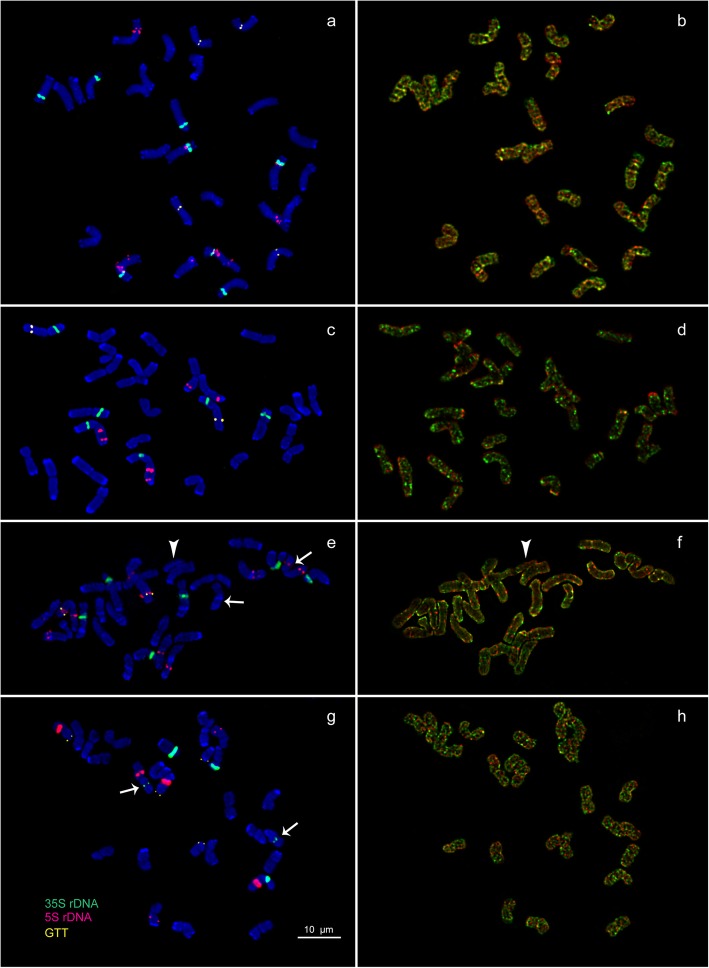
Chromosome spreads of the studied polyploid species. **a**, **b** *A. arundinaceus*, **c**, **d** *A. latifolia*, **e**, **f** *D. cespitosa* and **g**, **h** *D. flexuosa*. Merged fluorescent images after MC-FISH with 35S rDNA (green), 5S rDNA (red), oligo-(GTT)_9_ (yellow) **a**, **c**, **e**, **g** and sequential rapid MC-GISH with genomic DNAs of *D. sukatschewii* (red) and *H. lanatus* (green) **b**, **d**, **f**, **h**. DAPI chromosome staining - blue. Arrows point to chromosome rearrangements. Head of arrows point to a B chromosome. Scale bar - 10 μm

**Fig. 4:**
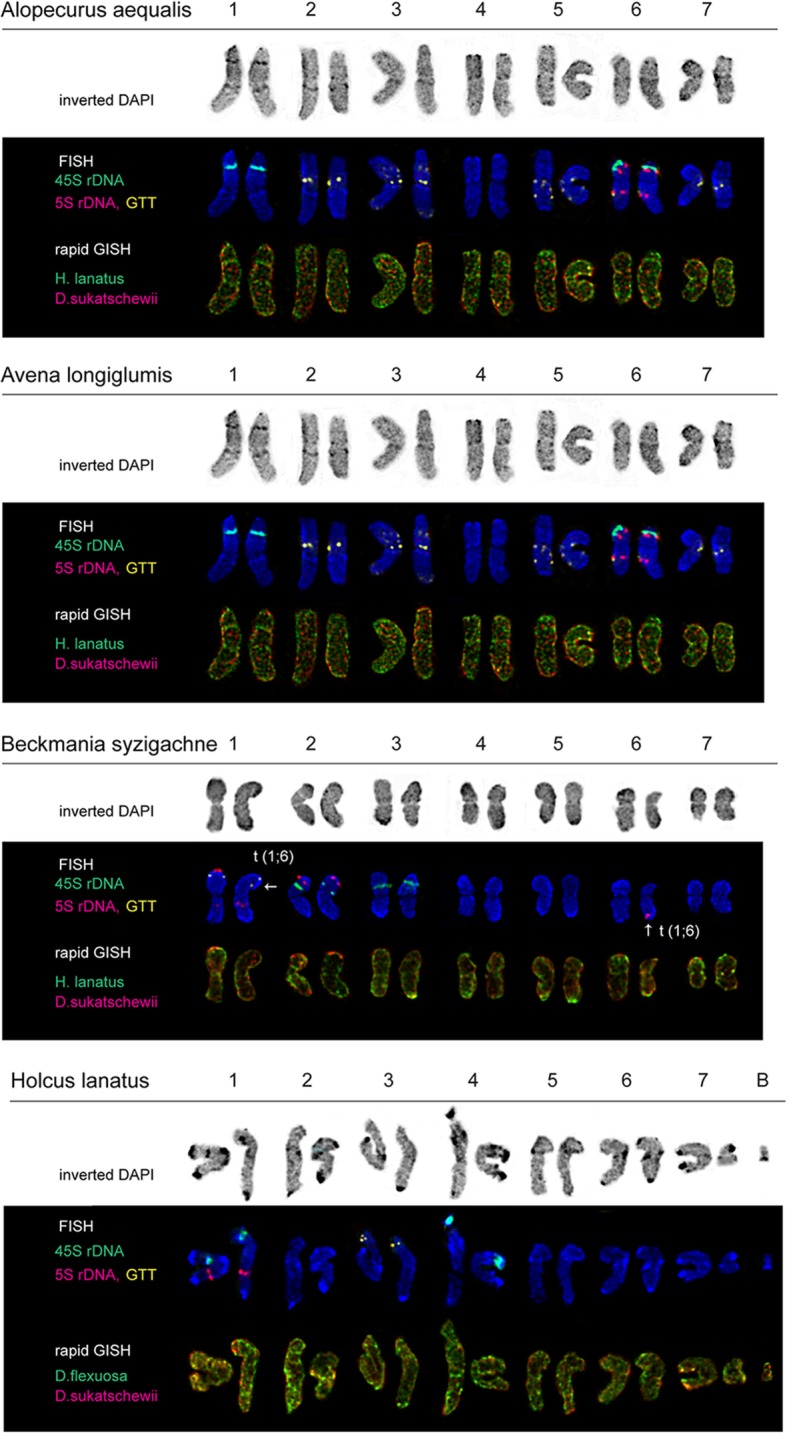
Karyotypes of *A. aequalis*, *A. longiglumis*, *B. syzigachne* and *H. lanatus*. Karyograms of the metaphase plates shown in Fig. 2 after DAPI-banding (inverted images), MC-FISH with the oligo-(GTT)_9_, 35S and 5S rDNA probes and also rapid MC-GISH with genomic DNAs of *D. flexuosa*, *D. sukatschewii* and *H. lanatus*. The correspondent probes and their pseudo-colours are specified in the left. Arrows point to chromosome rearrangements. B -B chromosome

**Fig. 5:**
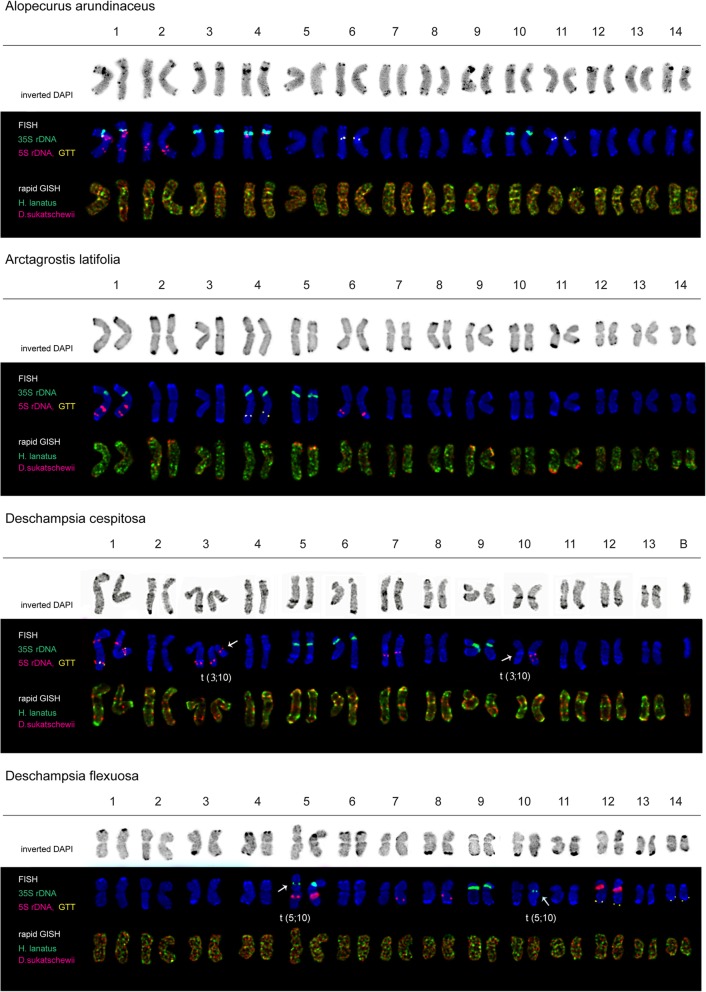
Karyotypes of *A. arundinaceus*, *A. latifolia*, *D. cespitosa* and *D. flexuosa*. Karyograms of the metaphase plates shown in Fig. 3 after DAPI-banding (inverted images), MC-FISH with the oligo-(GTT)_9_, 35S and 5S rDNA probes and also rapid MC-GISH with genomic DNAs of *H. lanatus* and *D. sukatschewii*. The correspondent probes and their pseudo-colours are specified in the left. Arrows point to chromosome rearrangements. B - B chromosome

The accessions of the studied grass species presented diploid 2n = 14 (*A. aequalis*, *A. longiglumis*, *B. syzigachne* and *H. lanatus*) and also tetraploid 2n = 28 (*A. arundinaceus*, *A. latifolia* and *D. flexuosa*) karyotypes with the basic chromosome number x = 7 except for *D. cespitosa* having 2n = 26 chromosomes. In several karyotypes of four *D. cespitosa* plants, one to three supernumerary small chromosomes with uncertain morphology were detected (Figs. 3e, f and 5). The number of these supernumerary chromosomes varied between the studied *D. cespitosa* plants as well as within the root meristem of each plant. In one karyotype of *H. lanatus*, a supernumerary chromosome was also revealed (Figs. 2i, j and 4).

Visual analysis showed that karyotypes of *A. aequalis*, *A. longiglumis*, *A. arundinaceus*, *A. latifolia, B. syzigachne*, *D. flexuosa* and *H. lanatus* contained metacentric and submetacentric chromosomes which were not very different in size and morphology (Figs. 2, 3, 4, and 5). In *D. cespitosa* karyotypes, metacentric, submetacentric and subtelocentric chromosomes were observed (Figs. 3e and 5).

### MC-FISH mapping of the (GTT)_9_ oligonucleotide, 35S and 5S rDNA

MC-FISH analysis revealed different patterns of chromosomal distributions of oligo-(GTT)_9_, 35S and 5S rDNA probes in karyotypes of the studied accessions summarized in Tables 1 and 2 (Supplementary information files).

In all examined karyotypes of the *A. aequalis* accession, MC-FISH analysis (performed for the first time) revealed large 35S rDNA hybridization sites in the secondary constriction regions (short arms) of chromosome 6. Sites of 5S rDNA were detected in the interstitial regions of chromosome pair 1 (long arms) and 4 (short arms). Small (GTT)_9_ sites were revealed in the subterminal regions of chromosome pair 2 (short arms) and 6 (long arms) (Figs. 2a and 4).

In all examined karyotypes of the *A. longiglumis* accession, 35S rDNA loci were reveled in the secondary constriction regions (short arms) of two chromosome pairs 1 and 6. Sites of 5S rDNA were detected in chromosome pair 6 (in the short arm adjacent to 35S rDNA loci and also in the proximal region of the long arm). The oligo-(GTT)_9_ probe produced signals on five chromosome pairs. Distinct signals were observed in the pericentromeric regions of chromosome pairs 2, 3 and 7. Besides, a number of minor sites were observed in chromosome pair 3 (in the interstitial and proximal regions of the short arms and in the subterminal region of the long arms) and chromosome pair 5 (along the long arms) (Figs. 2c and 4).

In all studied karyotypes of four plants of *B. syzigachne*, MC-FISH analysis (performed for the first time) revealed 35S rDNA loci in the secondary constriction regions (short arms) of two chromosome pairs 2 and 3. 5S rDNA sites were observed in the subterminal region of the short arms and in the interstitial region of the long arms of chromosome pair1, and also in the distal region of the short arms of chromosome pair 2. Small (GTT)_9_ sites were detected in the distal region of the short arms of chromosome pair 1 (Figs. 2e and 4).

In all studied karyotypes of one plant of the *B. syzigachne* accession, 35S rDNA loci were revealed in the secondary constriction regions (short arms) of two chromosome pairs 2 and 3. 5S rDNA sites were observed in the subterminal region of the short arm (in the hemizygote state) and in the interstitial region of the long arms of chromosome pair 1, in the distal region of the short arms of chromosome pair 2 and in the distal region of the long arm (hemizygote state) of chromosome pair 6. Small (GTT)_9_ sites were detected in the distal region of the short arms of chromosome pair 1 (Figs. 2g and 4).

In all studied karyotypes of the *H. lanatus* accession, MC-FISH analysis (performed for the first time) revealed 35S rDNA loci in the secondary constriction regions (short arms) of chromosome pairs 1 and 4. Sites of 5S rDNA were detected in the interstitial region of the long arms of chromosome pair 1. Small (GTT)_9_ sites were observed in the distal region of the short arms of chromosome pair 3 (Figs. 2i and 4).

In all examined karyotypes of the *A. arundinaceus* accession, MC-FISH analysis (performed for the first time) revealed large 35S rDNA sites in the secondary constriction regions (short arms) of chromosome pairs 1, 3, 4 and 10. Sites of 5S rDNA were visualized in the proximal region of the short (adjacent to the 35S rDNA site) and long arms of chromosome pair 1, in the interstitial region of the long arms of chromosome pair 2 and in the short arms of chromosome pair 4 (adjacent to the 35S rDNA site). Distinct (GTT)_9_ sites were detected in the pericentromeric regions of chromosome pairs 6 and 11 (Figs. 3a and 5).

In all studied karyotypes of the *A. latifolia* accession, MC-FISH analysis (performed for the first time) revealed large 35S rDNA sites in the secondary constriction regions (short arms) of chromosome pairs 1, 4 and 5. Sites of 5S rDNA were visualized in the interstitial and distal regions of the long arms of chromosome pair 1 and also in the distal regions of the long arms of chromosome pair 6. A small (GTT)_9_ site was detected in the distal regions of the long arms of chromosome pair 4 (Figs. 3c and 5).

In all examined karyotypes of the *D. cespitosa* accession, 35S rDNA loci were located in the secondary constriction regions (short arms) of chromosome pairs 5, 6 and 9*.* 5S rDNA sites were observed on chromosome pair 1 (in the interstitial regions of both short and long arms), chromosome pair 3 (in the distal region of the long arms and in the proximal region of the short arm (hemizygous state)), chromosome pair 7 (in the proximal region of the long arms) and chromosome pair 10 (in the proximal and interstitial regions of the long arm (hemizygous state)). Small (GTT)_9_ sites were revealed in the interstitial region of the long arms of chromosome pair 1 (Figs. 3e and 5).

In all examined karyotypes of the *D. flexuosa* accession, 35S rDNA loci were detected in the secondary constriction regions (short arms) of chromosome pairs 5 (a small site) and 9. Also, a small 35S rDNA locus was detected in the pericentromeric region of chromosome pair 10 (hemizygous state). Large 5S rDNA sites were detected in the interstitial region of the long arms of chromosome pair 5 and in the interstitial region of the short arms of chromosome pair 12. Besides, minor sites were revealed in the proximal regions of the long arms of chromosome pairs 7 and 8 (hemizygous state). Small (GTT)_9_ sites were detected in the subterminal regions of the long arms of chromosomes pairs 12 and 14 (Figs. 3g and 5).

### Chromosomal markers revealed by rapid MC-GISH

A rapid MC-GISH procedure with total genomic DNAs of *H. lanatus* and *D. sukatschewii* as DNA probes was performed on chromosomes of *A. aequalis*, *A. arundinaceus*, *A. latifolia*, *A. longiglumis*, *B. syzigachne*, *D. cespitosa* and *D. flexuosa*. On chromosomes of *H. lanatus*, a rapid MC-GISH procedure was performed with the use of total genomic DNAs of *D. flexuosa* and *D. sukatschewii* as DNA probes*.*

Multiple clustered hybridization signals (rapid MC-GISH markers) of *H. lanatus* were revealed in different positions on chromosomes of *A. aequalis* (Figs. 2b and 4), *A. arundinaceus* (Figs. 3b and 5), *A. latifolia* (Figs. 3d and 5), *B. syzigachne* (Figs. 2f, h and 4) and *D. cespitosa* (Figs. 3f and 5). Dispersed and few clustered hybridization signals of *H. lanatus* were observed along the chromosomes of *D. flexuosa* (Figs. 3h and 5)*.* Few small clustered (coinciding with the localization of 35S rDNA sites) and dispersed hybridization signals of *H. lanatus* were observed along the chromosomes of *A. longiglumis* (Figs. 2d and 4).

On chromosomes of *H. lanatus*, the rapid MC-GISH analysis revealed few small clustered and dispersed hybridization signals of *D. flexuosa* (Figs. 2j and 4).

Multiple clustered hybridization signals of *D. sukatschewii* were revealed in different positions on chromosomes of *A. aequalis* (Figs. 2b and 4), *A. arundinaceus* (Figs. 3b and 5), *B. syzigachne* (Figs. 2f, h and 4) and *D. cespitosa* (Figs. 3f and 5). Besides, few small hybridization signals of *D. sukatschewii* were detected on chromosomes of *H. lanatus* (Figs. 2j and 4) and *A. latifolia* (Figs. 3d and 5). Dispersed and small clustered hybridization signals of *D. sukatschewii* were observed along the chromosomes of *D. flexuosa* (Figs. 3h and 5)*.* Dispersed signals of *D. sukatschewii* were observed along the chromosomes of *A. longiglumis* (Figs. 2d and 4).

### DAPI-banding analysis

Chromosome staining with DAPI performed after FISH/GISH procedures revealed DAPI-banding patterns specific to the studied species accessions. Large (intense) DAPI-bands were observed in karyotypes of *A. arundinaceus*, *A. latifolia*, *D. cespitosa*, *D. flexuosa* and *H. lanatus* (mostly, in the pericentromeric and subterminal regions of chromosomes). Also, middle-sized (interstitial, proximal and distal) bands were revealed on chromosomes of *A. latifolia* (chromosome pair 12), *A. longiglumis* (chromosome pairs 1, 2, 4 and 5), *A. arundinaceus* (chromosome pairs 1, 3, 4, 8, 10 and 14), *D. cespitosa* (chromosome pairs 1, 3, 5, 8 and 12), *D. flexuosa* (pair 12), and *H. lanatus* (chromosome pairs 1 and 4). On the other chromosomes of the studied accessions, small bands were detected. B chromosomes found in karyotypes of *D. cespitosa* and *H. lanatus* possessed distinct DAPI-bands (Figs. 4 and 5).

### Karyotype analysis

Based on chromosomal morphology, DAPI-banding patterns, distribution of the oligo-(GTT)_9_, 35S and 5S rDNA probes as well as rapid GISH markers, chromosomes in karyotypes of the studied species were identified and the species karyograms were constructed (Figs. 4 and 5). The comparison of patterns of distribution of the examined molecular cytogenetic markers allowed us to reveal different chromosomal rearrangements in karyotypes of *B. syzigachne*, *D. cespitosa* and *D. flexuosa* (detailed in Figs. 4 and 5).

## Discussion

Within the *Aveneae*/*Poeae* tribe complex, most grass species were shown to have a basic chromosome number x = 7, though other basic chromosome numbers have also been found (http://www.tropicos.org/project/ipcn). Besides, a natural polyploid series as well as variation in ploidy level were revealed [4, 15, 27, 39]. Polyploidy is widespread in grass species, and it considered to play an important role in the evolution of vascular plants [53, 54]. The speciation within the *Aveneae*/*Poeae* tribe complex has been accompanied with episodes of polyploidy and intergeneric hybridization between the representatives of this tribe (especially, having hybrid or/and rearranged genomes) resulted in appearance of allopolyploid species [55–58]. Polyploidization events often seem to be associated with increases in vigor followed by adaptation of newly formed polyploids to novel conditions [55–57], so polyploids are able to colonize larger geographic ranges and/or occur in more habitats than related diploids [54]. The genera *Alopecurus, Arctagrostis*, *Beckmania*, *Deschampsia* and *Holcus* comprise widespread polyploid species and/or polymorphic forms which are highly tolerant to stressful and/or variable environmental conditions [6, 8–10, 55, 59]. The species *A. aequalis*, *A. arundinaceus*, *A. latifolia*, *B. syzigachne***,** *D. cespitosa*, *D. flexuosa*, *D. sukatschewii* and *H. lanatus* are predominant grasses within the pastures of the Arctic and sub-Arctic regions [6, 10, 11]. However, only scattered data on their distribution in the vast territory of Eurasia is currently available. In the present study, we constructed the integrated schematic maps of their occurrence in the northern, central and eastern parts of Eurasia based on the currently available data [2, 6, 45–52]. These maps indicate the vast areas with multi-species occurrence and also the regions (predominantly in the Far North and the Far East) where only certain grass species are now distributed. In these regions, shortage in forage resources could be recovered through the introduction of the other Arctic grassland species with high levels of adaptation and seed productivity as well as developing new valuable cultivars with the use of promising wild morphotypes [11–13]. The grassland accessions examined in the present study were sampled in different parts of the sub-Arctic tundra (North West and Far East regions and also the highlands with sub-Arctic mountain climate) characterized by harsh climate, shallow soils, permafrost, high levels of UV radiation, low participations, strong winds, etc. In plants grown under various abiotic environmental stresses, different cytogenetic abnormalities (mixo-, aneu- and polyploidy, chromosome rearrangements, variability in chromosome number, distribution of rDNA loci and other DNA repeats, appearance of B chromosomes, etc.) are especially common [27, 37], and this karyotype diversity is considered to be related to the plasticity of plant genomes [60, 61]. Despite the stressful natural environmental conditions, the cytogenetic analysis showed that the studied accessions presented normal diploid (2n = 2x = 14, *A. aequalis*, *A. longiglumis*, *B. syzigachne* and *H. lanatus*) and tetraploid (2n = 4x = 28, *A. arundinaceus*, *A. latifolia* and *D. flexuosa*) karyotypes with the typical for cereals basic chromosome number x = 7, except for the paleopolyploid *D. cespitosa* having 2n = 26 chromosomes. Our findings agreed with the cytological data reported earlier [36, 37, 62]. Particular, for *D. cespitosa* and some other *Deschampsia* species with 2n = 26, the analysis of meiotic chromosome behavior confirmed the basic number of chromosomes (*n* = 13) and also their diploid status [63]. This unusual for cereals chromosome number could be related to descending dysploidy, one of the most crucial routes of post-polyploid genome diploidization, which is described for several taxa of the *Aveneae/Poeae* tribe complex [64].

In some cells of the studied *D. cespitosa* accession and also in one metaphase plate of the *H. lanatus* accession, we detected supernumerary small chromosomes with uncertain morphology and distinct DAPI bands. The frequency of those supernumerary chromosomes varied between individual plants as well as within root meristem of each plant. They could be referred to B chromosomes because Bs are known to be extra karyotype components which are generally smaller than the normal chromosomes (A chromosomes), often possess heterochrornatic segments and exhibit non-Mendelian inheritance [65]. B chromosomes have previously been revealed in various grass species including *D. cespitosa* [37, 62, 65]. Their appearance in a karyotype is associated with genome instability though functional role of Bs is still not fully understood [65, 66]. In this study, the appearance of Bs in karyotypes of *D. cespitosa* and *H. lanatus* accessions could be related to environmental stress factors as a correlation between the presence of Bs in a karyotype and environmental conditions was described earlier [67, 68]. At the same time, the B chromosome detected in *H. lanatus* could be a fragment resulted from a break involving nearby chromosome 7 (which was smaller than its homolog). Considering that Bs have not previously been described in *H. lanatus*, further molecular cytogenetic studies of different accessions of this species are required.

Repetitive DNAs are major components of plant genomes which have high evolution rates and can lead to genome diversity [61, 69]. Knowledge of cytogenetic positions of specific repetitive sequences (chromosomal markers) provides information on genome structure differences which is important for analysis of the structural evolution of plant chromosomes [70]. Particularly, in karyotypes of vascular plants, DAPI staining, performed after FISH or GISH procedures, reveals AT-rich heterochromatin (which comprises highly repetitive DNA sequences) as strongly stained bands [71]. Clustered localization of highly repetitive DNA sequences (large distinct DAPI-bands) was observed on chromosomes of *A. arundinaceus*, *A. latifolia*, *D. cespitosa*, *D. flexuosa* and *H. lanatus* whereas in karyotypes of the other species, including *A. longiglumis*, we detected small DAPI-bands. The similar chromosomal distribution of constitutive heterochromatin was earlier described for different *Avena* species with the use of C-banding technique [30–32]. The comparison of the species distribution areas and their cytogenetic peculiarities indicated that karyotypes of two accessions from sub-Arctic mountain tundra (*H. lanatus* and previously described *D. sukatschewii* [37]) possessed larger DAPI-bands (constitutive heterochromatin,) compared to the other accessions. This could be related to different pathways of plant genome reorganization (probably, for adaptation to the extreme environments) which involved highly repeated DNAs.

Physical mapping of ribosomal 35S and 5S DNA on chromosomes of diploid and polyploid plant species provides information on the structural evolution of the chromosomes carrying these sequences [28, 29, 72], whereas nucleotide similarity among diploid and polyploid rDNA copies reveals some of their phylogenetic and genomic relationships [73]. In this study, the comparative karyotypic analysis of the diploid and tetraploid accessions of *Alopecurus* (*A. aequalis* and *A. arundinaceus*) revealed chromosomes with similar morphology and distribution patterns of 35S rDNA, 5S rDNA and DAPI-bands (e.g., chromosome 1 in *A. aequalis* and chromosome 2 in *A. arundinaceus*; chromosome 6 in *A. aequalis* and chromosome 10 in *A. arundinaceus*) which could be related to the allopolyploid origin of the *A. arundinaceus* genome. Interestingly, in both *Alopecurus arundinaceus* and *Avena longiglumis* (Al genome) karyotypes, we indicated one pair of chromosomes with the similar pattern of multiple rDNAs localization (large terminal 35S rDNA and distal 5S rDNA sites in the short arm and also interstitial 5S rDNA loci in the long arm). The similar chromosome pair was earlier described in diploid and polyploid *Avena* species with different types of the A genome [30–34], and it could be inherited from a common progenitor at a remote period. In karyotypes of *A. latifolia*, *H. lanatus* and *D. flexuosa*, we observed a large chromosome carrying a distal 35S rDNA site in the short arm and interstitial 5S rDNA loci in the long arm. The occurrence of multiple rDNA sites localized in specific chromosomes may have value in chromosome identification and elucidation of evolutionary relationships and also delineation of possible break point sites [29, 74].

Currently, microsatellite DNA sequences are widely used as FISH probes for cytogenetic studies as they are major components of many plant genomes [33, 34, 75]. Particularly, it has been recently determined that FISH with the oligo-GTT probe produces six constant signals located in the pericentromeric regions of three chromosome pairs of diploid A genome *Avena* species with minor interspecies differences in signal intensity [34]. These data indicate genomic variations among AA species and agree with the results of C-banding analysis [30–32] and Southern hybridization [76]. In the *A. longiglumis* accession studied here, FISH with the oligo-(GTT)_9_ probe revealed not only the six cluster signals mentioned above but also a number of minor signals which demonstrated intraspecific variability in chromosomal distribution of this microsatellite motif. Interestingly, on chromosomes of two species, *Avena longiglumis* and *Alopecurus arundinaceus*, clustered (GTT)_9_ signals were detected in the pericentromeric regions. In karyotypes of the other studied species, only small distal or subterminal (GTT)_9_ sites were observed. This agree with the molecular phylogenetic and cytogenetic data reported earlier [5, 34] and could be related to distant relationships between these species.

According to current molecular phylogenetic studies, the studied here grass species (except *A. longiglumis*) are included in chloroplast group 2 (*Poaeae* type) which is subdivided into two clades comprising genera 1) *Avenella*, *Deschampsia*, *Holcus* and 2) *Alopecurus*, *Arctagrostis, Beckmannia* [1]*.* In the present study, we used a rapid GISH approach to reveal common homologous DNA repeats in karyotypes of the studied species groups. We have previously reported that a rapid GISH procedure with genomic DNA of *D. cespitosa* revealed multiple large hybridization signals on chromosomes of *D. sukatschewii* (confirming their close relationships). We have also found that *D. sukatschewii* genome was rich in AT-heterochromatin [37]. Besides, the species from both *Deschampsia* and *Holcus* contain common DNA repeats CON1, CON2, COM1 and COM2 which are widespread in *Poaceae* [15]. In the rapid GISH assays performed in the present study, we used labelled genomic DNAs of *D. sukatschewii* and *H. lanatus* (chloroplast group 2) to reveal common DNA repeats on most studied species except *H. lanatus.* For *H. lanatus* chromosomes, we used genomic DNAs of *D. sukatschewii* and *D. flexuosa* as *D. flexuosa* differed karyotypically from *D. sukatschewii* and *D. cespitosa* [37]. The performed rapid MC-GISH analysis showed that species from both chloroplast groups possessed common DNA repeated sequences as clustered hybridization signals of genomic DNAs of both *H. lanatus* and *D. sukatschewii* were revealed in different positions on chromosomes of *A. aequalis*, *A. arundinaceus*, *B. syzigachne*, *D. cespitosa* and *D. flexuosa.* On chromosomes of *A. latifolia*, visual analysis revealed more hybridization signals of genomic DNA of *H. lanatus* (which therefore, indicated more genome similarities between these species) compared to genomic DNA of *D. sukatschewii*. At the same time, on chromosomes of *A. longiglumis*, which belongs to chloroplast group 1 (*Aveneae* type) [1, 5], we mostly observed dispersed hybridization signals of genomic DNAs of both *H. lanatus* and *D. sukatschewii*. Thus, our findings generally agree with the molecular phylogenetic data reported earlier [1, 5].

The genus *Beckmannia* comprises two perennial species, *B. eruciformis* and *B. syzigachne*. This genus has been subjected to several taxonomic revisions. Based on morphology, *Beckmannia* has been assigned to tribes *Phalarideae*, *Chloriideae*, *Beckmanniinae* and finally *Aveneae* (subtribe *Alopecurinae*) [77]. Until recently, molecular phylogenetic studies have not separated *Beckmannia* in a distinct lineage, tentatively leaving it within subtribe *Alopecurinae* [78–80] though support for potential separating *Beckmannia* and *Alopecurus* from each other has been provided [78]. Finally, however, Soreng et al. [1] has placed the genera *Alopecurus* and *Beckmannia* in two different subtribes (*Alopecurinae* and *Beckmanniina*). In support of these recent data, the molecular cytogenetic analysis performed in the present study did not reveal any karyotypic similarities between *B. syzigachne* and both diploid and tetraploid *Alopecurus* accessions. Nevertheless, further investigations of *Beckmannia* species based on different chromosomal and molecular markers are necessary to clarify the phylogenetic position of *Beckmannia* within the *Aveneae*/*Poeae* tribe.

Distribution patterns of the examined molecular cytogenetic markers in the studied here sub-Arctic accessions of *D. cespitosa* and *D. flexuosa* agreed with our previous results obtained for non-polar accessions [37]. Nevertheless, differences in number and size of some DAPI-bands as well as in localization of several chromosomal markers (mainly due to chromosomal rearrangements) were also observed. Molecular cytogenetic analysis of these species showed that their karyotypes differed significantly from each other. These results agreed with our previous findings indicating that *D. flexuosa* also had basic karyotypic differences with *D. antarctica*, *D. danthonioides*, *D. elongata*, *D. sukatschewii* and *D. parvula* [37]. According to the phylogenetic analyses inferred from nuclear ITS and plastid trnL sequence data, *D. flexuosa* is regarded a better suited to the genus *Avenella* [81]. However, rapid GISH assays with labelled genomic DNAs of both *D. sukatschewii* (in the present study) and *D. cespitosa* [37] detected clustered signals on chromosomes of *D. flexuosa* indicating the presence of common homologous highly repeated DNA sequences in their genomes.

It should be noticed that phylogenetic position of the genus *Deschampsia* within the family *Poaceae* is still controversial. According to Soreng et al. [1], *Deschampsia*, *Holcus* and *Vahlodea* were classified in the subtribe *Holcinae* which is not, however, monophyletic because *Holcus* and *Vahlodea* do not form a clade with *Deschampsia* in the plastid and nuclear ribosomal DNA trees [21, 22, 82]. In a parallel classification of grasses, *Holcinae* is treated as a synonym of *Airinae* [4]. Some recent authors render the genus *Deschampsi*a paraphyletic [83] or consider that *Deschampsia* would be better treated in its own monotypic subtribe [23]. Our findings agreed with the last of these observations as the comparative molecular cytogenetic analysis of *Deschampsia* species and *Holcus lanatus* did not reveal similarities in distribution patterns of the studied chromosomal markers.

In karyotypes of *B. syzigachne*, *D. cespitosa* and *D. flexuosa* accessions sampled in different parts of the sub-Arctic tundra (Far East and North West regions), chromosomal rearrangements were revealed. The presence of numerous chromosomal rearrangements in plant karyotypes is considered to be related to the genome plasticity [60, 61] and/or to speciation events [70, 84]. Accordingly, the process of genome evolution in these taxa could include chromosomal reorganization (chromosome interchanges, inversions, translocations) of the initial parental genomes.

Thus, the results of the present study provide unique information on distribution areas and cytogenomic structures of valuable Arctic and sub-Arctic pasture grass species from related genera of the *Aveneae*/*Poeae* tribe which revealed structural differences and also similar features in their karyotypes**.** The obtained results can be a basis for the further genetic and biotechnological studies.

## Conclusions

The peculiarities of distribution patterns of the examined chromosomal markers and also presence of common homologous DNA repeats in karyotypes of the studies species allowed us to verify their relationships. The revealed karyotypic similarities between *Alopecurus aequalis* and *Alopecurus arundinaceus* indicated the allopolyploid origin of the *A. arundinaceus* genome. The karyotype of *Beckmannia syzigachne* differed greatly from the studied *Alopecurus* accessions in support of the recent molecular phylogenetic studies which have placed the genera *Alopecurus* and *Beckmannia* into different subtribes within the *Poeae* tribe. The comparative molecular cytogenetic analysis of *Deschampsia* species and *Holcus lanatus* confirmed the recent molecular phylogenetic data which suggested not be classified *Deschampsia* and *Holcus* in one subtribe. Our findings are important for further genetic and biotechnological studies and also plant breeding progress.

## Supplementary informations

**Additional file 1: Table 1.** Localization of 35S rDNA, 5S rDNA and (GTT)_9_ sites on chromosomes of the studied diploid accessions

**Additional file 2: Table 2.** Localization of 35S rDNA, 5S rDNA and (GTT)_9_ sites on chromosomes of the studied polyploid accessions

## Data Availability

All data generated or analysed during this study are included in this published article and its supplementary information files.

## Abbreviations

MC-FISH: multicolour fluorescence in situ hybridization
rapid MC-GISH: rapid multicolour genomic in situ hybridization
rDNA: ribosomal DNA
sat DNA: satellite DNA
