# Supplementary material for: Molecular cytogenetics of valuable Arctic and sub-Arctic pasture grass species from the Aveneae/Poeae tribe complex (Poaceae)
Source: BMC Genet. 2019 Dec 4;20:92. doi: 10.1186/s12863-019-0792-2 (PMC6894191; doi:10.1186/s12863-019-0792-2)
Supplement: Supplementary file 1 — Additional file 1: Table 1. Localization of 35S rDNA, 5S rDNA and (GTT)9 sites on chromosomes of the studied diploid accessions [file 12863_2019_792_MOESM1_ESM.doc]

**Table 1. Localization of 35S rDNA, 5S rDNA and** **(GTT)_9_ sites** on chromosomes of the studied diploid accessions

| Species | Chromosome | | | | | | | |
| --- | --- | --- | --- | --- | --- | --- | --- | --- |
|  | arm | number | | | | | | |
|  |  | 1 | 2 | 3 | 4 | 5 | 6 | 7 |
| *A. aequalis* | S |  | GTTst |  | 5Sint |  | 35Ssc |  |
|  | L | 5Sint |  |  |  |  | GTTst |  |
| *A. longiglumis* | S | 35Ssc | GTTpc | GTTint  GTTprx  GTTpc |  |  | 35Ssc 5S adj | GTTpc |
|  | L |  |  | GTTst |  | GTTmlt | 5Sprx |  |
| *B. syzigachne* | S | 5Sst hz  GTTdst | 35Ssc  5Sdst | 35Ssc |  |  |  |  |
|  | L | 5Sint |  |  |  |  | 5Sdst hz |  |
| *H. lanatus* | S | 35Ssc |  | GTTdst | 35Ssc |  |  |  |
|  | L | 5Sint |  |  |  |  |  |  |

S - short chromosome arm; L - long chromosome arm; 35Ssc - 35S rDNA site localized in the secondary constriction region; 5Sint (st, prx, dst, adj) - 5S rDNA site localized in the interstitial (subterminal, proximal, distal, adjustment to 35S rDNA site) chromosome region; GTTpc (st, dst) - (GTT)_9_ sites localized in the pericentromeric (subterminal, distal) chromosome region; GTTmlt - multiple (GTT)_9_ sites localized along the long chromosome arm; hz - hemizygote state.
