# Supplementary material for: Molecular cytogenetics of valuable Arctic and sub-Arctic pasture grass species from the Aveneae/Poeae tribe complex (Poaceae)
Source: BMC Genet. 2019 Dec 4;20:92. doi: 10.1186/s12863-019-0792-2 (PMC6894191; doi:10.1186/s12863-019-0792-2)
Supplement: Supplementary file 2 — Additional file 2: Table 2. Localization of 35S rDNA, 5S rDNA and (GTT)9 sites on chromosomes of the studied polyploid accessions [file 12863_2019_792_MOESM2_ESM.doc]

**Table 2. Localization of 35S rDNA, 5S rDNA and (GTT)_9_ sites on chromosomes of the studied polyploid accessions**

| Species | Arm | Chromosome number | | | | | | | | | | | | | |
| --- | --- | --- | --- | --- | --- | --- | --- | --- | --- | --- | --- | --- | --- | --- | --- |
|  |  | 1 | 2 | 3 | 4 | 5 | 6 | 7 | 8 | 9 | 10 | 11 | 12 | 13 | 14 |
| *A. arundinaceus* | S | 35Ssc  5Sadj |  | 35Ssc | 35Ssc  5Sadj |  | GTTpc |  |  |  | 35Ssc | GTTpc |  |  |  |
|  | L | 5Sprx | 5Sint |  |  |  |  |  |  |  |  |  |  |  |  |
| *A. latifolia* | S | 35Ssc |  |  | 35Ssc | 35Ssc |  |  |  |  |  |  |  |  |  |
|  | L | 5Sint  5Sdst |  |  | GTTdst |  | 5Sdst |  |  |  |  |  |  |  |  |
| *D. cespitosa* | S | 5Sint |  | 5Sprx hz |  | 35Ssc | 35Ssc |  |  | 35Ssc |  |  |  |  |  |
|  | L | 5Sint  GTTint |  | 5Sdst |  |  |  | 5Sprx |  |  | 5S prx  5Sint hz |  |  |  |  |
| *D*. *flexuosa* | S |  |  |  |  | 35Ssc |  |  |  | 35Ssc | 35Ssc hz |  | 5Sint |  |  |
|  | L |  |  |  |  | 5Sint |  | 5Sprx hz | 5Sprx hz |  |  |  | GTTst |  | GTTst |

S - short chromosome arm; L – long chromosome arm; 35Ssc - 35S rDNA site localized in the secondary constriction region; 5Sint (st, prx, dst, adj) - 5S rDNA site localized in the interstitial (subterminal, proximal, distal, adjustment to 35S rDNA site) chromosome region; GTTpc (int, dst, st) - (GTT)_9_ sites localized in the pericentromeric (interstitial, distal, subterminal) chromosome region; hz - hemizygote state.
